# H1N1 vaccination and adults with underlying health conditions in the US

**DOI:** 10.1371/currents.RRN1132

**Published:** 2009-12-07

**Authors:** Edward Goldstein, Marc Lipsitch

**Affiliations:** ^*^Harvard School of Public Health and ^†^Dept of Epidemiology and Center for Communicable Disease Dynamics, Harvard School of Public Health

## Abstract

65% of fatalities from pH1N1 infections in a large US case series occur in adults with underlying health conditions other than pregnancy, but it appears that only relatively few high-risk adults will get vaccinated during the fall wave of pH1N1 transmission. There are several reasons for this problem; the most important is vaccine shortage. High risk adults (other than pregnant women) were not part of the initial, narrow priority cohort which included pregnant women and children ages 0.5-4; this is despite the fact that some of those high risk groups, such as adults with immunosuppressive conditions and possibly individuals with neurological disorders, have a relative risk for fatality (per capita) higher than pregnant women, and over 28-fold higher than healthy children under the age of 4. With more vaccine becoming available than needed in the initial priority cohort, a broader group which includes high risk adults and individuals under 24 becomes eligible for vaccine in many locations. Nonetheless, due to continuing high demand, high-risk adults face competition for vaccine from healthy individuals under 24; additionally, some locations specifically prioritize school students over high-risk adults. Finally, there is an issue of awareness and a shortage of specific channels that target high risk adults other than pregnant women and facilitate vaccine distribution among them in the US.

A recent CDC estimatesuggests that death toll from H1N1 is larger than previously thought [1], approximately 4000 deaths from April to October 2009. In a recent case series**[2]**, over 65% of fatalities among hospitalized H1N1 patients were adults with underlying health conditions other than pregnancy.   A previous study [3], with a higher proportion of children among the hospitalized patients than [2], found that 72% of hospitalized adults had an underlying condition other than pregnancy. Among individuals who died or were admitted to ICU in the same study, 67% had an underlying health condition. Other studies [4] [5] have found a high percentage of individuals with underlying medical conditions among the H1N1 related fatalities. In children about 70-80% of fatalities involved underlying conditions [6] (particularly neurological disorders [7]).

With limited information on H1N1 fatalities stratified by age and underlying conditions, we attempt to assess the relative risks of some high risk group for which data is available. Our data sources are [2] [8] [9] [10]. Also throughout this paper, a relative risk for death for a high-risk group is the ratio of the death rate (per capita) in this high-risk group over the death rate in the whole population.

Pregnant women constitute 1% of the US population [8]. The proportion of pregnant women among deaths in [2] is 5.1%. Thus an estimate of the relative risk for pH1N1 death for pregnant women in the US is 5.1.

Morbidly obese adults (BMI ≥ 40) constitute 4.8% of the adult US population and correspondingly 3.63% of the of the whole US population [10].  Data in [2] suggest that morbidly obese adults constitute 31.5% of fatal cases. Thus, the relative risk for death for morbidly obese adults may be estimated as 8.7.  

Data on the prevalence of immunosuppressing conditions have been assembled by the MIDAS High Risk Segmentation Group [9] from the 2006 National Health Interview Survey, the CDC HIV/AIDS surveillance 2007 report, the US Renal Data System, and the Organ Procurement and Transplantation Network, and kindly provided by Diane Wagener.  Immunocompromised adults represent 1.9% of the US population [9]. They represent 30.5% of all fatalities in [2]. Immunocompromised adults thus have an estimated 16-fold higher risk of death than the general population.

We have no data on prevalence of individuals with neurological (neurocognitive and neuromuscular) disorders, but adults with neuromuscular disorders represent 11.9% of all deaths in [2].  People with neurocognitive or neuromuscular disorders each represent 13.4% of deaths or ICU admissions in [3].

Children aged 0.5-4 (6-59 months) without underlying conditions make up about 6% of the US population. We have no data on their share among the H1N1-related fatalities. Children under 18 make up 13.9% of fatalities in [1], and 6.8% of fatalities in [2]. The number from [1] may be an overestimate as it makes certain assumptions on ascertainment of fatalities in children and adults; nonetheless we assume it to estimate children’s share among the fatalities. [6] suggests that only 20-30% of fatalities in children involve no major underlying conditions; the number in [2] is 2/8. We assume that 25% of fatalities in children involve no major underlying conditions. Finally we need to know the share of those aged 0.5-4 among the fatalities in healthy children. Even if we assume it is 100%, the relative risk for fatality of healthy children aged 0.5-4 (compared to the whole US population) is estimated as at most 0.58 (25% x 13.9%/6%); the estimated risk for adults with immunosuppressive conditions is 28 times that of healthy children aged 0.5-4. If we assume that at most half of all fatalities in healthy children occur in those aged 0.5-4, the relative risk for adults with immunosuppressive conditions compared to healthy children aged 0.5-4 becomes 56.

We want to point out that unlike the case of fatalities, children have the highest rates for H1N1-relatedhospitalizations (http://www.cdc.gov/vaccines/recs/acip/downloads/mtg-slides-oct09/12-2-flu-vac.pdf); moreover among children, the percentage of those not having major underlying conditions is higher for hospitalizations than for fatalities [2] [3] [6]. The high percentage of children among the hospitalizations, together with the perception that among all groups in the general population, children merit the most protection(http://www.flu.gov/individualfamily/vaccination/allocationguidance.pdf) were some of the reasons behind the high prioritization that healthy children received in the US.

**Vaccine distribution to high-risk adults: **Despite high relative risks for certain groups of adults with underlying health conditions, it appears that only a few of them will get immunized in time to be protected against the fall wave of H1N1 infections in the US.  With demand for vaccines exceeding supply, a number of jurisdictions, at least initially, have restricted vaccine distribution to a priority cohort that does not include high risk adults [11] [12] [13]. The ACIP’s recommended “Subset of Target Groups During Limited Vaccine Availability” includes pregnant women, caregivers for infants less than 6 months, health care workers at direct infection risk, children 6 months to 4 years, and high-risk children 5-18, but not high-risk adults. While we disagree with the choice to exclude high-risk adults from this “Subset of Target Groups,” we do not advocate changing it at this time. However, many jurisdictions are beginning to expand vaccination beyond this Subset, and more will do so as vaccine supplies grow.  In some locations, this expansion involves prioritizing school-age children over high-risk adults [14] [15] a choice that does not maximize the use of the vaccine to prevent fatal outcomes.  Evidently a more common strategy is to offer vaccine to a broader group that includes high risk adults and all individuals under 24 [16] [17] [18]. Inevitably, this creates competition for still-limited vaccine [19].

One possible way to resolve the discrepancy above is creation of a channel which will specifically target high-risk adults, increasing both their awareness and vaccination levels. For instance in the UK, GPs contact their priority patients and offer them vaccine [20]; also in the UK, people with underlying conditions are prioritized over children under 5 [21]. A similar strategy of health care providers focusing on patients with underlying conditions is being pursued in Oregon [22]. An alternative suggestion is to offer vaccines specifically for high risk individuals, perhaps opening clinics in large adult ambulatory care practices or in other settings where high-risk adults are likely to be present.  Such clinics might limit their offerings to individuals with high risk conditions, similar to campaigns in schools that are limited to school-age children [23] [14] [24]. As public health messaging expands beyond the initial “Subset of Target Groups,” we urge that special emphasis be placed on adults in documented high-risk groups, in an effort to maximize the impact of remaining vaccine delivery on pH1N1 mortality.

**Acknowledgment:** The MIDAS High Risk Segmentation Group (D. Wagener, R. Zimmerman, D. Lauderdale) kindly shared the data from [9].

**Funding Information:** This work was funded by the US National Institutes of Health Models of Infectious Disease Agent Study Cooperative Agreements 5U01GM076497 and 1U54GM088588 to ML for the Harvard Center for Communicable Disease Dynamics (ML,EG).

**Competing Interests:** ML has consulted for the Avian/Pandemic Flu Registry (Outcome Sciences), funded in part by Roche, and for Novartis Vaccines and Diagnostics.  EG declares no competing interests.
